# 

**Published:** 1875-09

**Authors:** 


Physiological Action of Gelsemia. By J. Ott, M.D., Demonstrator of Physiology in the University of Pennsylvania. 12 mo. pamphlet, pp. 17.

---

A Report on Trichinosis, as observed in Dearborn county, Indiana, in 1874. By George Sutton, M.D., Aurora, Indiana. 8 vo., pamphlet, pp. 23.

---

Steiger's Classified Catalogue of American, British, German, and French Periodicals in the Departments of Medical Sciences, Chemistry, Pharmacy, etc., June, 1875. To be had of E. Steiger, 22 Frankfort street, New York.

---

We have received the annual circulars and catalogues of the following Medical Colleges: Bellevue Hospital Medical College, New York; University of Nashville, Nashville, Tenn.; National Medical College of the Columbian University, Washington, D. C.

